# Understanding the Effect of Different Abiotic Stresses on Wild Marigold (*Tagetes minuta* L.) and Role of Breeding Strategies for Developing Tolerant Lines

**DOI:** 10.3389/fpls.2021.754457

**Published:** 2022-02-03

**Authors:** Ajay Kumar, Rahul Dev Gautam, Ashok Kumar, Satbeer Singh, Sanatsujat Singh

**Affiliations:** ^1^Academy of Scientific and Innovative Research, CSIR-HRDC, Ghaziabad, India; ^2^Division of Agrotechnology, Council of Scientific and Industrial Research-Institute of Himalayan Bioresource Technology, Kangra, India

**Keywords:** abiotic stresses, adaptability, genetic variability, physiology, QTL, ROS, wild marigold, essential oil

## Abstract

Wild marigold has a growing demand for its essential oil in the flavor and fragrance industries. It can be grown over a broad range of climates, but the changing climatic conditions lead to abiotic stresses, thus restricting its productivity. Abiotic stresses at elevated levels result in the reduction of germination, growth, and essential oil quality of wild marigold leading to heterogeneous and inferior grades of “*Tagetes* oil.” Drought, salinity, and heavy metal stress at elevated levels have common effects in terms of ROS formation, which are the major cause of growth deterioration in wild marigold. Temperatures above 35°C inhibit seed germination. Irradiance stress reduces the biomass and essential oil yield. Waterlogging adversely affects the survival of wild marigold in high rainfall regions. The application of plant nutrients (fertilizers) modulates the biomass and essential oil yield. Wild marigold employs multiple tolerance mechanisms to cope up with the adverse effects of abiotic stresses such as the increased activity of antioxidants to maintain cellular redox homeostasis, enhanced lipid peroxidation in the cell membrane to maintain cell wall architecture, production of secondary metabolites, and accumulation of osmolytes. In this review, we tried to understand how abiotic stresses affect wild marigold. Understanding the physiological changes and biochemical characteristics of stress tolerance will contribute to the development of stress-tolerant lines of wild marigold.

## Introduction

Wild marigold is a member of the *Asteraceae* family, the most prominent family among vascular plants (Cornelius and Wycliffe, 2016). Wild marigold is suitable for cultivation as monocrop or intercrop in both hills and plain areas (Singh et al., 2003). The essential oil obtained from wild marigold has the best value among all the species of this genus (Chopra et al., 1963). The market demand for *Tagetes* oil has increased due to its higher demand in the flavor and fragrance industry. The essential oil is also reported to have antimicrobial, insecticidal, antifungal, nematicidal, and allelopathic biological activities (Kumar et al., 2020). The farmers are showing interest in its cultivation and opting for it in their cropping system. High bee activity during flowering in wild marigold indicates that essential oil also helps in attracting the pollinators and ensures seed set due to its high fragrance. The annual production of its essential oil was ∼15 tons during 2016 and is continuously increasing per year. The global essential oil market is expected to expand with a compound annual growth rate (CAGR) of 8.6% from 2019 to 2025.

Wild marigold occurs in a broad range of climates worldwide and is native to North and South America (Cornelius and Wycliffe, 2016). It occurs naturally throughout the globe, including Europe, Asia, and Africa (Babu and Kaul, 2007). In India, it got naturalized in the Western Himalayas between 1,000 and 2,500 m altitudes. Himachal Pradesh is the number one producer state of wild marigold essential oil in India. The current estimate of its essential oil production in the world is 8–15 tons per annum (Cornelius and Wycliffe, 2016). The essential oil is traded internationally under HS Code 3301. The largest importers of “*Tagetes* oil” are Germany (worth USD 49,580), Taiwan (worth USD 2,160), and the United States (worth USD 997) (Bandana et al., 2018). The productivity of wild marigold varies significantly from season to season and over different locations. Most importantly, the unpredictable climatic variations/abiotic stresses are the utmost constraint for wild marigold production. Abiotic stresses are the climatic or soil conditions affecting the cellular homeostasis of plants, resulting in impaired growth and development (Mickelbart et al., 2015). The drought stress (Mohamed et al., 2000, 2002; Farahani et al., 2009; Cicevan et al., 2014, 2016; Zulfiqar et al., 2020; Babaei et al., 2021), temperature stress (Forsyth and Staden, 1983), irradiance/shade stress (Kumar et al., 2014), waterlogging (Singh et al., 2003), salinity stress (Sahay and Patra, 2013; Sayyed et al., 2014; Chrysargyris et al., 2018; Pazcel et al., 2018; Moghaddam et al., 2019), heavy metal (HM) stress (Patel and Patra, 2014; Cid et al., 2016; Pazcel et al., 2018), and application of nutrient fertilizers (Walia and Kumar, 2021b) all affect the morphophysiological performance and secondary metabolism (essential oil) of wild marigold.

The formation of reactive oxygen species (ROS) increases under drought, salinity, and HM stress conditions in wild marigold and is the major cause of growth deterioration. Enhanced accumulation of ROS in the plant cell degrades the photosynthetic machinery of the plant disrupting the chloroplast membrane and, therefore, reducing the photosynthetic activity (Sihem et al., 2020). Wild marigold employs multiple mechanisms to cope up with the destructive effect of abiotic stresses like increasing the activity of antioxidative enzymes such as catalase (CAT), polyphenol oxidase (PPO), ascorbate peroxidase (APX), and guaiacol peroxidase (GPX) (Babaei et al., 2021). The other non-enzymatic compounds having antioxidant properties which increase under drought and salinity stress conditions are carotenoids, phenols, ascorbic acid, and tocopherol. Wild marigold starts to accumulate low-molecular-weight compounds (osmolytes), such as proline, polyamines, and glycine betaine, under adverse environmental conditions as a defense mechanism to reduce osmotic stress in the plant cell (Moghaddam et al., 2019; Babaei et al., 2021). Elevated levels of abiotic stress induce an imbalance between ROS production and antioxidative defense system resulting in oxidative stress, which disrupts the cells and chloroplast membrane. Membrane lipid peroxidation results in the production of malondialdehyde (MDA) and under the presence of ROS, its production occurs more rapidly. The production of secondary metabolites in plants under abiotic stresses depends on the physiological and developmental conditions (Mahajan et al., 2020). Besides providing a variety of valuable natural products, secondary metabolites help to protect plants against pathogenic attacks and environmental stresses. Abiotic stress signals increase the expression of transcription factor (TF) families, e.g., Apetala2/ethylene-responsive factor (AP2/ERF), WRKY [highly conserved amino acid sequence WRKYGQK and the zinc-finger-like motifs Cys (2)-His(2) or Cys(2)-HisCys], basic helix-loop-helix (bHLH), basic leucine zipper (bZIP), MYB (myeloblastosis gene family of TFs), and NAC [No Apical Meristem (NAM), *Arabidopsis* transcription activation factor (ATAF), and Cup Shaped Cotyledon (CUC)] TFs, which are involved in biochemical pathways of secondary metabolites (Tuteja, 2007; Meraj et al., 2020). TFs act as a mediator, and they receive stress signals and direct the downstream defense gene expression. The interaction of TFs and stress signal contributes to the accumulation of particular secondary metabolites under stressed conditions (Meraj et al., 2020).

The secondary metabolites of wild marigold are monoterpenes, sesquiterpenes, flavonoids, and thiophenes. The essential oil of wild marigold contains four main compounds, namely, Z-β*-*ocimene, dihydrotagetone, limonene, tagetones (E and Z), and ocimenones (Kumar et al., 2020; Walia et al., 2020). *Tagetes* oil with 42–47% Z*-*β-ocimene is considered to be a high grade in the international market with respect to quality (Cornelius and Wycliffe, 2016) while tagetone is valuable for biocidal activity (Walia et al., 2020; Babaei et al., 2021). Despite the vast information on wild marigold morphology, chemical, and genetic diversity, very limited information is available in literature about the consequences of environmental factors on growth and associated essential oil metabolic changes. In this review, we have tried to understand the effect of different abiotic stresses on wild marigold growth and yield. The development of stress-tolerant and stable varieties of wild marigold is a necessity. Assessing genetic variability for stress tolerance in natural populations and making selections for stress tolerance traits can be effectively utilized to identify stress-tolerant lines of wild marigold which can be used as parental lines for reconstitution of new populations to counter various abiotic stresses under different climatic conditions.

## Effects of Different Abiotic Stresses on Wild Marigold

**FIGURE 1:**
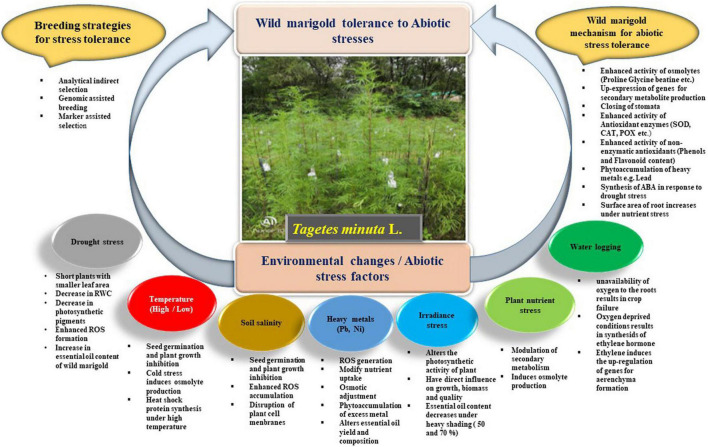
Schematic representation of different abiotic stresses affecting morphophysiological, biochemical, and secondary metabolism of wild marigold and plant strategies to tolerate adverse environmental conditions along with breeding approaches for stress tolerance.

The production of wild marigold faces numerous challenges due to different abiotic stresses. The schematic presentation of different abiotic stress factors on wild marigold along with plant mechanisms to cope up with adverse environmental conditions is given in Figure 1. The possible role of breeding approaches for abiotic stress tolerance is discussed accordingly for each stress condition.

### Effect of Drought Stress on Wild Marigold

Drought stress affects the physiology and biochemical responses of wild marigold and essential oil yield (Babaei et al., 2021). Water deficit during the vegetative phase results in shorter plants with smaller leaf areas to reduce water use. The morphological traits of wild marigold, such as plant height, stem diameter, leaf fresh weight, flower fresh weight, stem fresh weight, leaf dry weight, flower dry weight, and stem dry weight, decrease with the elevated drought conditions (Babaei et al., 2021). There is a decrease in the vegetative dry matter of aromatic plants under drought stress (Shahri et al., 2013). The physiological parameters of wild marigold, such as relative water content % (RWC), chlorophyll content, and carotenoid content, decrease with the intensifying water limitations at four levels of 100, 75, 50, and 25% field capacity (Babaei et al., 2021). The photosynthesis under drought is limited by CO_2_ concentration in the chloroplast, determined by stomatal and mesophyll conductance (Ouyang et al., 2017). In water deficit conditions, stomatal conductance is affected by leaf anatomical traits (e.g., size and density of stomata), which varies while acclimating to the stressed environment, affecting the transpiration rate. Studying leaf anatomical variations among natural populations of wild marigold can help improve the leaf structural characteristics required for tolerance under water deficit conditions in wild marigold.

Drought-induced reduction in wild marigold growth is due to the formation of ROS in cellular compartments of chloroplast, peroxisome, and mitochondria under severe drought stress. The ROS starts to accumulate in the plant cell which results in degradation of membranes leading to lipid peroxidation (Chen et al., 2000), protein degradation (Jiang and Zhang, 2001), and nucleic acid damage, ultimately resulting in cell death (Hagar et al., 1996; Munné-Bosch and Peñuelas, 2003). ROS in the plant cell can degrade the photosynthetic machinery through disruption of the chloroplast membrane and, therefore, reduce the photosynthetic activity (Sihem et al., 2020). Membrane lipid peroxidation results in the production of MDA, and under the presence of ROS, its production occurs more rapidly. Electrolyte leakage and MDA content increases under drought stress in wild marigold have been recently reported by Babaei et al. (2021).

The enhanced concentration of ROS in cellular compartments is controlled by the antioxidant defense system that modulates the intracellular concentration and sets the cellular redox status. The antioxidative enzymes which are associated with drought stress tolerance in wild marigold are CAT, APX, and GPX, and the activity of all these enzymes increases under intensifying water limitations of 100, 75, 50, and 25% field capacity (Babaei et al., 2021). The production of other compounds, such as carotenoids, phenols, ascorbic acid, and tocopherol having antioxidant properties, increases with increasing levels of drought stress (Chaeikar et al., 2020), and the plant also starts to accumulate low-molecular-weight compounds (osmolytes), such as proline, polyamines, and glycine betaine, under elevated drought conditions (Cicevan et al., 2016). Osmolytes protect cell structure and function by delaying the dehydration damage and maintaining cell turgor under drought stress (Taiz and Zeiger, 2006). Osmolytes also help to improve the carbohydrate partitioning and metabolism during flowering and ultimate final yield. The proline content under drought stress in wild marigold increases up to 58% at 25% field capacity when compared with control (100% field capacity) (Babaei et al., 2021). Proline accumulation is reported to be under monogenic control in barley (Kueh and Bright, 1981). Accordingly, the heritability of such traits will be high and can be used as effective selection criteria.

In a study, while analyzing the use of somaclonal variations to select the drought-tolerant plants of wild marigold, Mohamed et al. (2000) used mannitol as an osmotic stress agent. Shoot clumps of wild marigold were developed on MS media (containing callus growth media) and then subculture on callus growth media containing different concentrations of mannitol (60 and 80 mM) for different durations (3, 6, and 9 months). When grown in greenhouse conditions for 2 months, one clone developed from shoot clumps selected from the media containing mannitol exhibited a tolerance up to 90 mM. This tolerant clone had higher soluble sugars and proline content than non-stressed clones. Furthermore, this clone showed higher accumulated biomass and higher relative growth than other regenerated and control plants. The procedure can be used as a screening technique for evaluating natural populations and selecting drought-tolerant plants. The selection of drought-tolerant genotypes is one of the leading research focuses to increase the yield under water deficit conditions (Chaves and Davies, 2010) for the conservation of natural resources (of water) and to meet the future demand of xeriscaping (Cicevan et al., 2016). Anatomical changes in the leaf, stem, and root of a plant can also be used as successful indicators of drought stress (Oliveira et al., 2018). Increasing water deficit levels enhanced the reduction in root vascular area, shoot cortex area, leaf thickness, and cortex has been reported for the related species of genus “*Tagetes*.”

Plant mechanisms to survive during water stress conditions include increased root biomass and length, reduced shoot growth, or altered leaf orientation to escape water limitations (Zulfiqar et al., 2020). Drought stress reduces the yield of wild marigold, possibly by a reduction in the leaf area (through wilting/rolling) during severe stress. It reduces the absorption of photosynthetically active radiations (PAR) by plant canopy and thus prevents the plant from accumulating new dry matter. Also, drought stress may limit the essential oil yield of wild marigold by reducing the harvesting index when a brief period of stress coexists during the critical developmental stage (flowering) (Earl and Davis, 2003). Drought stress during reproductive growth decreases the interval from seed germination to pollen shed. A recent study indicated a drastic reduction in yield due to drought stress during flowering in wild marigold (Mohamed et al., 2002). Therefore, it may be concluded that water deficit reduces vegetative growth, and as a result, plant switches to flowering, thus ultimately decreasing productivity.

Assessing variations in natural populations of wild marigold for these parameters will give insight into genetic variations for these traits for making effective selections. Similarly, some of the root traits that also need to be studied to improve crop tolerance under drought stress include total root length and total root biomass (Wasaya et al., 2018), which contribute to drought stress tolerance in plants. Abscisic acid (ABA) is a prime signal factor (plant hormone) activated in response to dehydration. It plays a significant role in drought avoidance by bringing about stomatal closure, reducing leaf expansion, and promoting root growth. These parameters may help to screen out stress-tolerant lines in the wild marigold. Therefore, an analytical breeding approach through the indirect selection of highly heritable stress-responsive traits could be the best strategy for genetic improvement for drought tolerance.

### Temperature Affects the Achene Germination of Wild Marigold

Seed germination is one of the primary stages affected by stress due to temperature fluctuations and directly impacts ultimate production in terms of crop stand. The optimum temperature lies between the minimum and maximum temperature limits where the highest germination percentage may be obtained within the shortest time (Mayer and Poljakoff-Mayber, 1975). In wild marigold, germination is wholly inhibited at a temperature of 35°C due to thermoinhibition, while 100% germination was evident when achenes were transferred to 25°C (Forsyth and Staden, 1983). Thermoinhibition may be used as a form of seed pretreatment, enabling rapid completion of germination on return to temperatures conducive to germination (Heydecker, 1977). This has been demonstrated for wild marigold achenes (Forsyth and Staden, 1983). When achenes are imbibed at 25°C, germination takes place over several days. When the achenes are first imbibed at 35°C for 24 h, 100% germination is recorded within 24 h of transfer to 25°C (Hills et al., 2001). Genetic variability for such traits can ensure better crop stands under temperature stress conditions and may contribute to biomass yield. Thus, assessing genetic variability among collections over different geographical locations followed by selection for best fit germplasm under stress conditions could be a prior important breeding strategy for improvement.

### Irradiance Stress Affects the Biomass and Essential Oil Composition of Wild Marigold

Solar radiations have a direct influence on the photosynthetic activity of the plant. Irradiance (flux of radiant energy per unit area) is an important environmental factor that directly affects plant growth, plant adaptation, reproduction, distribution, and metabolite composition (Keller and Lüttge, 2005; Guidi et al., 2008). While assessing the effect of irradiance stress in wild marigold, Kumar et al. (2014) evaluated four different levels of shades (0, 20, 50, and 70%) and three plant spacing geometries (45 × 30, 45 × 45, and 60 × 45 cm) on growth, biomass, and quality characteristics of the plant. Plants grown in 25% shading produced taller plants compared with those which were grown in full sunlight. The total number of branches and the spread of the plant decreased with increased shading. The 45 × 45 cm plant spacing results in the highest plant height and branches. The 45 × 30 cm plant spacing and 25% shading result in higher biomass of fresh leaves, stem, and flower biomass comparable with open light conditions. Heavy shading (50 and 70%) reduces the essential oil content in both leaves and flowers. Wild marigold occurs both as forest undergrowth and open patches in the hills resulting in essential oils of different grades. From a cultivation point of view, access to complete sunlight conditions will benefit crop productivity. However, assessment of variability in natural populations under shade conditions can give insights about plant growth and essential oil quality in wild marigold.

### Effect of Waterlogging on Survival of Wild Marigold

Wild marigold faces water logging problems especially in high rainfall areas (Singh et al., 2003). The unavailability of oxygen to the roots during waterlogged conditions is the primary reason for crop failure restricting the uptake of water and nutrients of soil in wild marigold. Genetic improvement for waterlogging tolerance in wild marigold requires prior knowledge on the genetics of traits, source of tolerance, and selection strategy in natural populations of wild marigold under waterlogged conditions. Ethylene changes the plant tolerance mechanism under deprived oxygen situations by inducing the expression of certain genes which are responsible for aerenchyma formation. Ethylene also induces the gene-associated enzymes of glycolysis, and fermentation pathways have been reported for wheat under waterlogged conditions (Alamgir and Uddin, 2011). Ethylene initiates flooding escape strategies through activation of the ERFVII TFs SNORKEL1 and 2 in deepwater rice, which in turn enhance internode elongation to escape hypoxia through the restoration of above-water gas exchange (Hattori et al., 2009).

There is no information available in literature regarding the waterlogging-induced oxidative stress in wild marigold (tolerance/avoidance). However, earlier reports on other crops, such as rice, wheat, and maize, suggested that waterlogging tolerance is a polygenic trait associated with several quantitative trait loci (QTLs). Since it is a complex trait and depends on many factors, such as growth stage, soil temperature, soil topography, and nutrient availability. An analytical approach through the indirect selection of component traits contributing to a low heritable target trait-like biomass yield in flooded conditions could be effective for genetic improvement.

### Effect of Salinity Stress on Wild Marigold

Soil salinity affects various physiological and yield contributing characters of the wild marigold including seed germination (Moghaddam et al., 2019). It also exerts negative effects on enzymatic and photosynthetic activity (Lee et al., 2013). The salinity tolerance during seed germination and early growth of wild marigold is important for establishing wild marigold in saline conditions. An increase in salinity concentrations causes a decrease in seed germination percentage (Delgado et al., 2016). Recently, Moghaddam et al. (2019) showed that the germination percentage of wild marigold decreased with increasing concentrations of sodium chloride and potassium nitrate (0–500 mM). The lowest germination percentage was recorded at 200 mM concentration of both the salts. There was a complete inhibition in the germination of wild marigold achenes above 200 mM concentrations for both (NaCl and KNO_3_) salts. These experiments indicate that above a tolerance limit of the species (200 mM in case of wild marigold), salinity can cause complete inhibition of seed germination.

The salinity induces many physiological and biochemical processes in wild marigold by accumulating low-molecular-weight solutes (osmolytes) such as proline. It is reported that the proline accumulation in wild marigold increased with increasing salinity levels (0, 50, 100, and 200 mM) of both NaCl and KNO_3_ (Moghaddam et al., 2019). Osmolyte accumulation helps in stabilizing the cellular structures of membranes, lipids, and proteins and scavenging free radicals during the formation of ROS under salt-induced oxidative stress (Sharma and Kumar, 2015). Total soluble sugar content and soluble protein also increased in a concentration-dependent manner. Thus, the content was highest at 200 mM concentrations of both the salts (Moghaddam et al., 2019). Salinity-induced oxidative stress results in ROS formation (Rejeb et al., 2014), and to cope with the harm caused by ROS, wild marigold evolved a defense system that limits ROS formation and accelerates their removal by the antioxidant defense system.

The activity of antioxidative enzymes, such as superoxide dismutase (SOD), POD, and CAT, increases with increasing salinity levels in wild marigold. The SOD activity in seedlings of wild marigold was higher under NaCl salinity than KNO_3_-induced salinity. The CAT activity showed almost similar trends of increase in both the salts, while peroxidase activity increased with increased concentrations of both the salts. The POD activity was higher in basal levels (50 and 100 mM) of NaCl concentrations (Moghaddam et al., 2019). Wild marigold is adapted to a wide range of climates due to higher tolerance for salts (e.g., 200 mM for NaCl and KNO_3_), pH, and exchangeable Na^+^ % in the soil; thus, it is considered as a potential crop for salt-affected regions (Sahay and Patra, 2013). Further genetic improvement for salt tolerance in this crop could be achieved following a recurrent selection approach utilizing landraces and germplasm collections of natural populations. However, there is a lack of available research on wild marigold germination, accumulation of osmolytes, antioxidant enzyme activities, and variability among natural populations for these traits under salinity stress conditions. The existing studies indicate considerable variability for response to salinity stress and need to be investigated in germplasm collections to identify potential lines for salinity stress tolerance.

### Heavy Metal Stress and Phytoaccumulation Potential of Wild Marigold

Wild marigold produces essential oils and accumulates HMs (Gautam and Agrawal, 2017; Pazcel et al., 2018). Thus, the species can extract hazardous metals from the environment, while it also provides economic benefits to the growers (Danh et al., 2010). Wild marigold accumulates lead (Pb), making it a potential candidate for the phytoextraction of this HM. The metal hyperaccumulator plant species can tolerate a higher concentration of a particular metal with less toxic impact (Kumar and Sharma, 2020). In an experiment, Sosa et al. (2016) observed that wild marigold plants collected from five different sites of a former battery recycling plant region with Pb contaminated soil accumulate Pb in the leaves while Pb was absent in the essential oil of the plant. Wild marigold responds to Pb pollution by modifying nutrient uptake mechanisms (root exudates), thus increasing the solubility/micronutrient uptake from the polluted soil. The variability in response to Pb among individuals from natural populations of wild marigold was recently studied in terms of exposed and unexposed populations (Pazcel et al., 2018). This study suggests that wild marigold has tolerance and accumulation potential for Pb. The interpopulation showed significantly higher survival rates than intrapopulation. The Pb concentration in roots and stems is higher irrespective of populations. The antioxidant activity in terms of the ferric-reducing ability of plasma (FRAP) reduces in both populations with a slight increase in interpopulation. A reduction in the soluble sugar content was recorded for intrapopulation while the same increased in interpopulation. Plants of both populations do not show any significant difference in lipid peroxidation (MDA content). The aerial biomass was higher in intrapopulation under Pb toxicity while the chlorophyll and carotenoid contents were higher (8.9 mg g^–1^/10.3 mg g^–1^ and 2.7 mg g^–1^/2.7 mg g^–1^, respectively) in both populations under Pb toxicity compared with control. The most dominant compound in essential oil for interpopulation reported was dihydrotagetone, while intrapopulation showed greater diversity in essential oil chemotypes. Phytoextraction potential in both natural and exposed populations of wild marigold confers a high variability between individuals suggesting that the selection process is the primary requisite to obtain tolerant cultivar when exposed to HM stress.

Wild marigold is reported to have higher plant growth and essential oil yield when grown in different textured soils (50 soil: 50 sludge) treated with varying concentrations of HM-rich tannery sludge (Patel and Patra, 2014). Tannery sludge at the highest concentration (100%) is inhibitory for soil microbial biomass and activity of urease enzyme. The activity of soil microbial biomass carbon, dehydrogenase, and acid/alkaline phosphatase increases in a concentration-dependent manner to the sludge in soil. The metal accumulation in the root tissue is higher than the shoot tissue, while no metal was detected in essential oil, suggesting wild marigold reduces metal toxicity by root absorption. A co-cropping system has been applied (Xu et al., 2013) to improve the phytoremediation of *Sedum alfredii*. They have recorded the higher growth and metal accumulation potential of hyperaccumulator plant species. The higher growth in hyperaccumulator species is due to synergistic effects while sharing the rhizosphere with low metal accumulator species (Wu et al., 2007). An experiment on two potential phytoextractor plants (*Bidens pilosa* L. and *Tagetes minuta* L.) co-cropped with lettuce growing on agricultural Pb-polluted soils (Cid et al., 2016) suggested that wild marigold produced significantly higher biomass in co-cropping. Also, the Pb accumulation in wild marigold increases significantly with co-cropping.

Nickel (Ni) plays a vital role in nitrogen (N) metabolism as a biologically essential nutrient, but it becomes toxic to the plants at higher concentrations. The effect of Ni and vermicomposting on plant growth, essential oil composition, and mineral element accumulation of wild marigold suggests that the growth of wild marigold with vermicomposting can be used as a strategy for rehabilitation and Ni phytoremediation (Chand et al., 2015). The high concentration of Ni in leaves than roots of wild marigold suggests that it can be used as a potential crop for Ni phytoremediation. Research on HM accumulation by wild marigold suggested a high variability in the extraction efficiency of individuals. Chronic exposure to HMs may result in tolerant ecotypes that arrest the harm produced by the metal. Variability for HM tolerance among and within populations was observed in response to HMs (Meyer et al., 2010), suggesting that selection will be effective for developing HM tolerant lines.

### Effect of Plant Nutrients on Wild Marigold

Plant nutrients, e.g., N and sulfur (S), have shown a considerable effect on wild marigold production and its essential oil content (Walia and Kumar, 2021a). Higher biomass and essential oil yield were recorded with increasing use of N and S fertilizers. Physiological characters, such as stomatal density, reduced with increasing levels of S (0, 20, 40, and 60 kg/ha) and N (0, 60, 90, and 120 kg/ha), while growth and yield parameters, such as plant height, number of branches/plant, leaf and flower biomass, stem biomass, total biomass, and essential oil yield, increased with the fertilizer dose. Furthermore, there is a need for the selection of lines that are not responsive to N and S as the cultivation of wild marigold is mostly performed in unutilized, degraded, and barren lands.

## Secondary Metabolism of Wild Marigold Under Abiotic Stress Conditions

**FIGURE 2:**
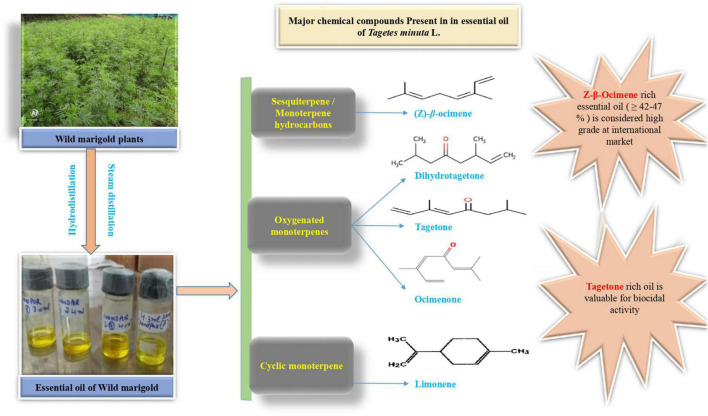
Major chemical compounds present in the essential oil of wild marigold.

The enhanced accumulation of secondary metabolites under abiotic stress conditions has been recently observed in wild marigold by Babaei et al. (2021). An increase in essential oil percentage has been reported but with a corresponding decrease in shoot yield. Therefore, the proportion of essential oil content to total biomass increases, although there is an overall decrease in biomass and essential oil content (Farahani et al., 2009). The secondary metabolites of the wild marigold include monoterpenes, sesquiterpenes, phenols, flavonoids, and thiophenes. The primary metabolites (carbohydrates, amino acids, lipids, and nucleotides) act as a precursor of secondary metabolites which do not contribute to plant growth and developmental processes. Instead, secondary metabolites have an important role in plant defense against abiotic stresses (Mahajan et al., 2020). The aroma of the wild marigold plant is due to the presence of “terpenoids,” which are synthesized in plants through the mevalonic acid (MVA) pathway in the cytoplasm and 2-methylerythritol 4-phosphate (MEP) pathway either in chloroplast or plastid. Terpenoids are the naturally occurring organic compounds derived from a five-carbon compound called as an isoprene unit. Isoprene units combine to form monoterpenes (10 carbon compounds) and sesquiterpenes (15 carbon compounds), which are the major secondary metabolites of wild marigold. Terpenes provide protection to the plant under abiotic stress-induced oxidative stress. Under the phenolic category, phenol and flavonoid are the major secondary metabolites of wild marigold produced mainly through the shikimic acid pathway, which is a major link between primary and secondary metabolism. The phenolic compounds slow down the formation of ROS and contribute to HM chelation. An increase in active substances under abiotic stresses is due to the high accumulation of reduced NADPH^+^ H^+^ (Mandoulakani et al., 2017), biosynthesis of specific plant hormones (Babaei et al., 2021), and upexpression of corresponding genes involved in the natural product biosynthesis (Mahajan et al., 2020). TFs perceive stress signals and direct downstream defense gene expression. Six major TF families including AP2/ERF, WRKY, bHLH, bZIP, MYB, and NAC have been reported to regulate the secondary metabolism under abiotic stress conditions (Meraj et al., 2020). The essential oil production under abiotic stresses contributes to plant defense and also protects it from biotic stresses. The average percent volatile oil content in wild marigold is around 0.3% (Kumar, 2019), which can be extracted either by hydrodistillation or steam distillation (Kumar et al., 2020). The essential oil of wild marigold contains four main constituents: Z-β*-*ocimene, dihydrotagetone, limonene, tagetones (E and Z), and ocimenones (Kumar et al., 2020; Walia et al., 2020), which are depicted in Figure 2.

The other aromatic compounds in turn are synthesized from these base compounds. The variation in essential oil yield and composition under different abiotic stresses is depicted in Table 1.

**TABLE 1:** Variation in essential oil yield and composition of wild marigold under different abiotic stress conditions.

S. No.	Abiotic stress condition	Evaluation	Essential oil content	Major metabolites	Effect on chemical composition	References
1.	**Drought stress**					
	Decrease in soil field capacity from 100 to 40%	Tolerant clone Non-tolerant clones Seed grown plant	Reduced to 49% for drought tolerant clone Reduced to 71% for non–drought tolerant Reduced to 71% for seed grown plant	Monoterpenes: *trans* and *cis*-tagetone, Limonene, dihydrotagetone	Altered the content of some oil components, e.g., sabinene	Mohamed et al. (2002)
	Decrease in Field Capacity (100, 75, 50, and 25%)	Morphological, physiological and phytochemical responses	Did not show a considerable effect on the essential oil content	Dihydrotagetone was the main constituent in all essential oil samples	Drought induces new constituents included 1,8-cineole and germacrene D	Babaei et al. (2021)
2.	**Irradiance stress** (Shade levels: 0, 25, 50, and 75%)	Leaf essential oil, flower essential oil and total essential oil	Increases up to 25% shade level and then declined with decrease in light intensity	Dihydrotagetones, tagetone, (*Z*)*-*β*-*ocimene and ocimenone	The ocimene and dihydrotagetones concentrations in leaves decrease with a decrease in irradiance while tagetone and ocimenone concentrations increases at 50 and 70% shading	Kumar et al. (2014)
3.	**Heavy metal stress** (Pb toxicity)	Phytoextraction potential of lead (Pb) on wild marigold leaves, growing near a battery recycling plan	The essential oils extracted by hydrodistillation did not have any detectable Pb	*cis*-tagetone, dihydrotagetone and verbenone	Pb concentration in leaves, increasing the content of β*-*ocimene and α-thujone	Sosa et al. (2016)
		Variability in terms of lead (Pb) Phytoextraction of wild marigold from Polluted Soils	Change in the composition of the essential oils associated with the increase of Pb in leaves was also observed	volatile composition of its essential oil that was associated with the activation of defense genes	Sabinene, limonene, β*-*ocimene, β*-*citral, and verbenone increases	Pazcel et al. (2018)
		Nickel (Ni) Toxicity	Changes in chemical composition of essential oil indicates that the application of Ni and vermicomposting had significant impact on the quality of *T. minuta* oil	Dihydrotagetone, tagetone and ocimene	Dihydrotagetone, tagetone and ocimene in *Tagetes minuta* oil were significantly decreased by the application of higher level of Ni	Chand et al. (2015)
4.	**Plant nutrients**	Nickel (Ni) and Sulfur (S) application	Essential oil yield increases with application of S and Ni	*Z-*β*-*ocimene, dihydrotagetone, tagetone, and ocimenone	*Z-*β-ocimene increases with higher N and sulfur doses While dihydrotagetone decreases with higher N dose	Walia and Kumar (2021a)

The ocimene and dihydrotagetone concentrations in leaves decrease with a decrease in irradiance, while tagetone and ocimenone concentrations increase under heavy shading of 50 and 70% when compared with control (Kumar et al., 2014). Pb concentration in leaves increases the concentration of α-thujone and β*-*ocimene (Sosa et al., 2016) suggesting the possible involvement of these compounds in Pb-induced stress tolerance.

*In vitro* screening of drought tolerance in wild marigold suggests that tolerant clones have higher essential oil percentages and yield during drought conditions. Under greenhouse conditions, decreasing the soil field capacity (from 100 to 40%) resulted in a reduction of essential oil yield by 49% in drought-tolerant clones compared with 71% in non-tolerant clones (Mohamed et al., 2002). The main components of the essential oil in drought-tolerant plants were monoterpenes (*trans*- and *cis*-tagetone together 52–64%). The effect of water stress on the essential oil yield of palmarosa (*Cymbopogon martinii* var. *motia*) revealed a decrease in essential oil content under drought conditions (Fatima et al., 2005). On similar lines, water deficit stress decreases the essential oil content in wild marigold, while it increases the essential oil percentage. The plants express stress tolerance or avoidance through the process of acclimation and adaptation evolved through natural selection. The traits which contribute to plant survival (e.g., roots grow deeper during drought) under stress are of great importance as these traits display functional conservation of the species. The substantial genetic variability for water stress tolerance in the genus “*Tagetes*” (ornamental) has been reported among different cultivars (Cicevan et al., 2016).

Recently, Babaei et al. (2021) reported that the essential oil content was not affected by drought conditions (100, 75, 50, and 25% field capacity). Essential oil of wild marigold mainly consisted of oxygenated monoterpenes, which were dihydrotagetone (65.3–75.3%) and (*Z*)-tagetone (35.8–40.9%). Monoterpene hydrocarbons in terms of (*E*)*-*β-ocimene were between 11.6 and 18.5%, while *E-*ocimenone, *Z-*ocimenone, and limonene were the other compounds in essential oil. Under elevated drought conditions, the oxygenated monoterpenes increase while monoterpene hydrocarbons slightly decrease. The β-pinene and carvone were not present at the higher drought level, while 1–8 cineole and germacrene D were the compounds that were observed under the severely stressed condition suggesting the possible role of these compounds in drought tolerance.

Application of plant nutrients, such as N and S, modulates the essential oil yield, and their compositions in wild marigold have been recently reported by Walia and Kumar (2021a). The application of 120 kg N/ha and 40 kg S/ha dose resulted in a higher essential oil yield of 50.08 and 18.27% compared with the control. The concentration of major compounds altered with the fertilizer dose. The (*Z*)*-*β-ocimene increases in a dose-dependent manner and was highest at a higher N dose of 120 kg/ha suggesting the possible role of plant nutrients in quality essential oil production in wild marigold. Furthermore, less information is available in literature regarding the effect of abiotic stresses on the essential oil yield and composition of wild marigold which needs to be studied. Simple field screening techniques and superior performances of the wild marigold genotypes for stress tolerance indicators could be successfully utilized to develop stress-tolerant lines.

## Effect of Crop Practices, Seasonal Variations, and Geographical Locations on Essential Oil Constituents of Wild Marigold

Natural populations of wild marigolds exhibit a wide range of essential oil constituents that vary from one individual plant to the other in the same season. Studies on the effect of crop practices and seasonal variations in essential oil composition of wild marigold (Singh et al., 2003) suggest that essential oil of the desired grade can be produced based on crop practice. The ocimene-rich essential oil can be obtained during the winter season, while dihydrotagetone, tagetone, and ocimenone rich oil can be obtained during autumn, summer, and autumn-winter, respectively. Effective selection for the quality of essential oil under particular stress conditions will require repetitive trials under similar stress conditions to establish the quality of essential oil over seasons. Wild marigold is a widely adapted plant and grows in different geographical regions of the world. The chemical composition of essential oil has a difference in chemical profile due to variation in geographical locations, plant part used, method of distillation, and growing conditions (Table 2).

**TABLE 2:** Essential oil composition as affected by different geographical locations and altitudes (world).

S. No.	Country	Geographical coordinates	Plant part used	Altitude (amsl)	Method of distillation	Average annual rainfall (mm)	Type of farming	Essential oil content	Major component	References
1	Turkey (Dörtyol, Hatay)	36° 49′ N, 36° 17′ E	Leaves and flower	750 m	Hydrodistillation	40	Dry	1.8%	*Trans-*β-ocimene (45.92%) verbenone 32.68%	Bahadirli (2020)
2.	Madagascar (Antananarivo)	18.87° S, 47.50° E	Leaves and flower color type	1,200 m	Steam distillation	1,084	Dryland	0.10–0.17%	(*Z*)-tagetenone (trace–26.7%) (*E*)-tagetenone (trace–31.3%) α-muurolene (trace–36.5%)	Ramaroson-Raonizafinimanana et al. (2009)
3.	Argentine (Province of Chaco)	27.42° S, 59.02° W	Leaves and flower	272 m	Steam distillation	760	Dryland	–	β-ocimene (45.4%)	Chamorro et al. (2008)
4.	Egypt	26.82 N, 30.80° E	Vegetative phase	500 m	Hydrodistillation	20	Dry	–	Dihydrotagetone (34.3 and 54.1%)	Senatore et al. (2004)
5.	South Africa (Alice, Eastern Cape)	32.79° S, 26.83° E	Vegetative phase	1,720 m	Hydrodistillation	494	Dry	–	*cis-*β-ocimene (50.9%)	Senatore et al. (2004)
6.	United Kingdom (Wolston)	52.37° N, 1.39° W	Vegetative phase	76 m	Hydrodistillation	410	Dry	–	*cis-*β-ocimene (32.0%)	Senatore et al. (2004)
7	Iran (Mahan, Kerman Province)	25.55° N, 53.26° E	Leaves, Flower and seed	1,755 m	Hydrodistillation	142	Dry	0.9% from leaves, 0.5% from seeds and 0.7% from flower	Dihydrotagetone (45.9%) in leaves, Benzoic acid esters (33.5%) in seeds and *trans-*ocimenone (27.0%) in flower	Moradalizadeh et al. (2013)
8.	Yemen	15.34° N, 44.19° E	Leaves	2,200 m	Hydrodistillation	127	Dry	–	(*Z*)-ocimenone (15.9%) and (*E*)-ocimenone (34.8%)	Ali et al. (2014)
9.	Rwanda	1.94° S, 29.87° E	Flower, branch and whole plant	950 m	Keiser-Lang apparatus	1,016	Dryland	0.8% in flower and 0.7% in leaves	Dihydrotagetone in leaves and (Z)-β-ocimene in flower	Chalchat et al. (1995)
10	Zambia	12.82° S, 28.21° E	Equal amounts of leaves and flowers	1,295 m	Hydrodistillation	900	Dryland	1.3%	Dihydrotagetone (30%) and (Z)-β-ocimene (23.6%)	Chisowa et al. (1998)
11	Hungary	47.62° N, 19.05° E	Flower	86 m	Hydrodistillation	600	Dry	0.50–1.10%	(Z)-β-ocimene (41%)	Hethelyi et al. (1986)
12	Brazil (Fortaleza)	3.73° S, 38.52° W	Flowers and inflorescence	21 m	Steam distillation	1042	Dryland	–	Dihydrotagetone (69.7%)	Craveiro et al. (1988)
13	North America	–	Whole plant	–	Hydrodistillation	767	Dryland	–	(Z)-β-ocimene (40.4%)	Bansal et al. (1999)
14	Uganda	0.34° N, 32.62° E	Fresh plant material	1240 m	Hydrodistillation	1,240	Rainfed	0.40%	*Trans-*ocimene (15.90%)	Kyarimpa et al. (2014)

The essential oil content decreases in dry farming, while essential oil content increases at higher altitude. So, the combined approach of growing improved planting material (stress-tolerant lines) along with good agro technological practices could be a better strategy in the dry regions.

In India, the variations in chemical profile due to different geographical locations are summarized in Table 3.

**TABLE 3:** Essential oil composition as affected by different geographical locations and altitudes (India).

S. No.	Location	Geographical coordinates	Plant part used	Altitude	Method of distillation	Average annual rainfall (mm)	Type of farming	Essential oil content	Major oil components	References
1	Himachal Pradesh (Palampur)	32.11° N, 76.53° E	Leaves and Inflorescence	1,220 m	Hydrodistillation	1,578	Rainfed	0.68%	(Z)-β-ocimene (52.01%) in flowers and dihydrotagetone (84.85%) in Foliage	Kumar et al. (2020)
2.	Himachal Pradesh (Kinnaur)	31.56 N, 78.12° E	Fresh aerial parts	2,637 m	Hydrodistillation	816	Dryland	0.79%	(Z)-β-ocimene (56.34%)	Walia et al. (2020)
3.	Himachal Pradesh (Sihunta, Chamba)	32.30 N, 76.07° E	Fresh aerial parts	489 m	Hydrodistillation	1,978	Rainfed	0.71%	(Z)-β ocimene (39.94%)	Walia et al. (2020)
4.	Uttarakhand (Silogi)	29.99° N, 78.57° E	Fresh aerial parts	1,850 m	Hydrodistillation	1,327	Rainfed	0.62%	(Z)-β-ocimene (48.45%)	Walia et al. (2020)
5.	Manipur (Senapati)	25.32° N, 94.15° E	Fresh aerial parts	2,500 m	Hydrodistillation	1,200	Rainfed	0.72%	(Z)-β-ocimene (52.43%)	Walia et al. (2020)
6	Lucknow	26.50° N, 80.50° E	Leaf, capitula, Whole plant	120 m	Hydrodistillation	990	Dryland	–	dihydrotagetone (32.0%) in whole plant, (Z)-tagetenones (31.2%) in flower and dihydrotagetone (50–60%) in capitula	Bansal et al. (1999)
7.	Hyderabad	26.89° N, 80.98° E	Dried mature fruits	243.8 m	Hydrodistillation	136	Dry	0.50%	(Z)-β-ocimene (36.8%)	Kaul et al. (2005)

The essential oil content increases with the higher altitude have been reported recently by Walia et al. (2020) while studying the variability in the chemical composition of wild marigold essential oil from different locations of Himalaya. The increase in the essential oil content at higher altitudes may be due to the response of the plants to produce more secondary metabolites under stress to cope up with adverse conditions and also there is the retention of essential oil in the plants under low temperature. Altitudinal variations affect the terpenoid biosynthetic pathway, and the oxygenated monoterpenes are higher at low altitudes while sesquiterpenes increase with the altitude.

## Selection of Morphological Traits Contributing to Biomass Enhancement in Wild Marigold

Leaves and flowers are the major contributors of biomass and essential oil yield in wild marigold (Rathore et al., 2018). Based on the review of literature, various attempts have been made to increase the biomass and essential oil yield of wild marigold through row spacing, N levels, phosphorous levels, irradiance stress, plant spacing, and weed management strategies (Singh et al., 2008; Kumar et al., 2014; Sharma et al., 2016; Zulfiqar et al., 2020; Walia and Kumar, 2021b). If the selections are made for higher biomass under optimum environmental conditions, at least some of these selected lines are expected to perform well under stress conditions. With this hypothesis, an assessment of variations for morphological traits in a wide range of germplasm lines representing natural populations collected from different geographical locations can be made. Aerial biomass in wild marigold at 100% flowering is a critical factor contributing to essential oil production (Rathore et al., 2018) and morphological traits, namely, plant height, number of branches, leaf number, and leaf size collectively constitute the aerial biomass. The correlation studies for these traits will help to understand the phenotypic architecture of wild marigolds to maximize biomass production under different stress conditions. Wild marigold has often-cross pollinated breeding behavior (Kumar et al., 2020), and collections of diverse origins are expected to be adapted to abiotic stress conditions to variable extent. Germplasm of wild marigold accessions representing collections from geographically diverse locations can contribute to increasing diversity for the stress tolerance traits and leads to enhanced genetic variation in the reconstituted populations. The data can be recorded for the morphological traits, namely, plant height, branches, leaf number, leaf length, leaf width, number of leaflets, leaflet length, leaflet width, and aerial biomass at the time of 100% flowering by selecting competitive plants from each accession line. Furthermore, the multivariate clustering of data can help organize the accessions collected from distinct environmental conditions (locations) into different phenotypic groups which may be categorized as superior, intermediate, and inferior based on significant differences for aerial biomass in comparison to the population mean (μ). This methodology may provide the key component traits for indirect selection in a genetic improvement program for higher biomass. Selection for the abovementioned morphological traits contributing to enhanced biomass is a critical strategy in maximizing production even in stressful environments. Therefore, the inheritance of morphological characteristics, such as plant height, branching, and leaf number, which are in general polygenic and greatly influenced by environmental conditions, need evaluation under stress environments at different growth stages of the plant to identify stress-tolerant lines.

## Role of Genomic-Assisted Breeding Approach for Abiotic Stress Tolerance in Wild Marigold

Exploration of the genomic resources, such as simple sequence repeat (SSR) markers or microsatellites and single nucleotide polymorphism (SNPs), is essential for mapping the specific genes or QTL of interest, including genes to abiotic stress tolerance in wild marigold. Based on the abiotic stress condition, a QTL can impart significant additive effects on account of genotype × environment (G × E) interaction (Collins et al., 2008). Identification of potential parental groups with contrasting responses to particular abiotic stress is a critical step for the development of mapping populations of wild marigold. Based on the breeding behavior of wild marigold (often cross-pollinated), the development of inbred lines through repeated selfing will be feasible for stress tolerance studies. High density genotyping using several SSR markers and accurate phenotyping of wild marigold mapping populations segregating for abiotic stress tolerance traits over locations and seasons could allow researchers to identify QTL-hotspots for abiotic stress tolerance traits. Mapping studies in wild marigold will help in the identification of loci that are responsible for heritable variations in phenotypic traits. Linkage mapping can be performed in wild marigold based on standard linkage mapping using seedlings from controlled crosses to produce full-sib progeny or pedigree-based mapping designs using half-sib progeny (one male pollinate different females). An alternative approach for mapping stress-related traits is through genome-wide association study (GWAS). In this approach, unrelated samples are characterized phenotypically under uniform field conditions for the evaluation of traits of interest and then genotyped through next-generation sequencing (NGS)-based whole-genome resequencing. Mapping abiotic stress-related traits in wild marigold populations (full-sib, half-sib, and F1 association mapping) will be more feasible in early stage seedlings where environmental conditions can be carefully controlled. Apart from high marker density and advanced platforms for phenotyping and genotyping, optimized statistical models for genomic sequencing and heredity of stress-related traits of wild marigold can contribute to the identification of such QTL-hotspots and maximize the precision of selection for multiple traits in early generations (Crossa et al., 2017; Dias et al., 2018; Voss-Fels et al., 2019). Furthermore, the functional genomic approach in wild marigold may act as a powerful tool for identifying candidate gene(s) involved in imparting tolerance to different abiotic stresses (Langridge et al., 2006). At present, the transcriptome data of wild marigold are not available in literature, which needs to be studied for new insights at the transcriptional level to understand the molecular basis of abiotic stress tolerance at different developmental stages. The technique can be utilized to develop functional markers based on traits that express differentially at the time of induced stress conditions in wild marigold. Natural populations of wild marigold form the requisite base material for carrying out these studies.

## Conclusion and Future Perspective

Abiotic stresses pose major constraints for the cultivation and production of wild marigold. Different abiotic stresses have commonalities, for instance, they affect plant survival, growth, and essential oil yield of the plant. Salinity, HM, and drought at elevated conditions result in the production of ROS, which is the major cause of growth deterioration under oxidative stress. There is an alteration in the activity of antioxidant enzymes (e.g., SOD, POD, POX, CAT, GPX, FRAP, and PPO) and non-enzymatic oxidants (e.g., total phenolic and flavonoid content) of wild marigold under these abiotic stress conditions. The MDA content increases under the abiotic stress condition which is a measure of lipid peroxidation of the cellular membrane. All the physiological parameters, namely, chlorophyll content, photosynthetic rate, stomatal conductance, and respiration rate are altered during stressed conditions. The leaf area index, water use efficiency, osmolyte regulation, and essential oil content are also affected under stressed conditions. There is also a reduction in seed germination, root growth, shoot growth, and plant biomass for these abiotic stresses. Similarly, high-temperature stress inhibits the seed germination due to thermoinhibition, while irradiance stress alters the growth and physiological responses of wild marigold and the quality of its essential oil. The abiotic stresses modulate the secondary metabolic pathways of wild marigold resulting in chemotypic variations in the essential oil composition of the plant. The modulation in secondary metabolism results in heterogeneous and inferior grades of essential oil in different abiotic stress conditions. The physiological traits, such as the early phenology of wild marigold, may help select genotypes exhibiting abiotic stress avoidance. Wild marigold is reported to have phytoaccumulation potential for Pb (HM). This study should also be focused on the development of elite selections more responsive to Pb accumulation.

A better understanding of the morphological, physiological, and biochemical responses of wild marigold to different abiotic stresses may help in the screening of elite genotypes in the germplasm of natural populations for the identification of abiotic stress-tolerant lines. The use of contrasting lines (tolerant and susceptible for each of the stress parameters) as parents in the hybridization program will lead to the development of mapping populations segregating for the particular stress. Evaluation of mapping populations across multiple locations and multiple seasons and screening of molecular markers in the mapping populations will result in the identification of molecular markers that closely map to specific genes or QTLs governing abiotic stress-related traits. This information will contribute to the systematic improvement of wild marigold through marker-assisted selection in reconstituted populations. The strategy aims at maximizing selection accuracy for the identification of breeding lines for abiotic stress tolerance in wild marigold.

## Author Contributions

AjK contributed to conceptualization, manuscript writing, and literature collection. RG contributed to literature collection. AsK contributed to important suggestions. StS contributed to create graphics and editing. SnS contributed to planning, monitoring, and editing. All authors contributed to the article and approved the submitted version.

## Conflict of Interest

The authors declare that the research was conducted in the absence of any commercial or financial relationships that could be construed as a potential conflict of interest.

## Publisher’s Note

All claims expressed in this article are solely those of the authors and do not necessarily represent those of their affiliated organizations, or those of the publisher, the editors and the reviewers. Any product that may be evaluated in this article, or claim that may be made by its manufacturer, is not guaranteed or endorsed by the publisher.

## Abbreviations

ROS: reactive oxygen species
QTL: quantitative trait locus
CAT: catalase
APX: ascorbate peroxidase
GPX: guaiacol peroxidase
PPO: polyphenol oxidase
MDA: malondialdehyde
POD: peroxidase.
